# Long-term survival analysis after endoscopic stenting as a bridge to surgery for malignant colorectal obstruction: comparison with emergency diverting colostomy

**DOI:** 10.6061/clinics/2020/e2046

**Published:** 2020-11-02

**Authors:** Rodrigo Corsato Scomparin, Bruno Costa Martins, Luciano Lenz, Luiza Haendchen Bento, Carlos Sparapam Marques, Adriana Safatle-Ribeiro, Ulysses Ribeiro, Sergio Carlos Nahas, Fauze Maluf-Filho

**Affiliations:** I Divisao de Endoscopia, Instituto do Cancer do Estado de Sao Paulo (ICESP), Universidade de Sao Paulo, SP, BR; II Divisao de Cirurgia, Instituto do Cancer do Estado de Sao Paulo (ICESP), Universidade de Sao Paulo, SP, BR

**Keywords:** Malignant Colonic Obstruction, Emergency Surgery, Colonic Endoscopic Stent, SEMS, Tumor Seeding

## Abstract

The use of colorectal self-expanding metal stents (SEMS) as bridge therapy for malignant colorectal obstruction was first reported more than 20 years ago. However, its use remains controversial.

**Objective:**

In this study, we aimed to compare the long-term survival of patients with potentially resectable malignant colorectal obstruction who had undergone colorectal SEMS placement and emergency surgery.

**Methods:**

This study was a retrospective analyses. Patients who received treatment between 2009 and 2017 were included. According to the eligibility criteria, 21 patients were included in the SEMS group and 67 patients were included in the surgical group..

**Results:**

The majority of the patients in the SEMS group were female (57.1%), whereas the majority of those in the surgical group were male (53.7%). The median follow-up time was 60 months for both groups with the same interquartile range of 60 months. There was no difference in the overall survival rate (log rank *p*=0.873) and disease-free survival rate (log rank *p*=0.2821) in the five-year analysis. There was no difference in local recurrence rates (38.1% *vs*. 22.4%, *p*=0.14) or distant recurrence rates (33.3% *vs*. 50.7%, *p*=0.16) in the SEMS and the surgical groups. Technical and clinical success rates of endoscopic stenting were 95.3% and 85.7%, respectively. There were no immediate adverse events (AEs). Severe AEs included perforation (14.3%), silent perforation (4.7%), reobstruction (14.3%), and bleeding (14.3%). Mild AEs included pain (42.8%), tenesmus (9.5%), and incontinence (4.76%). The limitations of this study was retrospective and was conducted at a single center.

**Conclusions:**

No differences in disease-free and overall survival rates were observed in the five-year analysis of patients with resectable colorectal cancer who had undergone SEMS placement or colostomy for the treatment of malignant colorectal obstruction. Patients in the SEMS group had a higher rate of primary anastomosis and a lower rate of temporary colostomy than did those in the surgery group.

## INTRODUCTION

Annually, more than 600,000 patients are diagnosed with colorectal cancer worldwide, leading to mortality in approximately half of these patients (1,2). It is estimated that approximately 25% of colorectal cancer patients with advanced-stage disease present with colorectal partial or complete obstruction (3).

Colorectal self-expanding metal stents (SEMS) have been extensively investigated as a bridge therapy for acute malignant colorectal obstruction. SEMS placement may be followed by elective surgical treatment once the patient’s clinical condition improves (4-7). A few systematic reviews and meta-analyses have reported that this strategy has reduced postoperative morbidity rates, including those of temporary and permanent colostomy (8-14).

Although the use of colorectal SEMS as a bridge therapy for malignant colorectal obstruction was first described more than 20 years ago (12), the therapeutic strategy remains controversial. There are concerns about the disease-free and long-term survival rates in patients with potentially treatable diseases. It was hypothesized that SEMS could induce micro-perforation because of seeding of malignant cells in the peritoneum, potentially increasing the risk of local cancer recurrence (15). Some researchers have reported higher rates of long-term recurrence (6,16,17) and significantly lower overall survival rates in the SEMS group (18). However, recent studies have described indirect evidence demonstrating that SEMS does not negatively impact survival (19-24).

## OBJECTIVES

In this study, we aimed to compare the long-term survival of patients with a potentially resectable malignant colorectal obstruction who had undergone colorectal SEMS with that in patients who had undergone emergency surgery.

The primary objective was to compare the overall survival and disease-free survival rates between the endoscopic and surgical groups. The secondary objectives were to compare the technical success, clinical success, re-intervention, and adverse event (AE) rates associated with stent placement, as well as to evaluate the permanent colostomy rates.

## MATERIAL AND METHODS

The data used in this retrospective study were extracted from medical records to compare patients who had undergone placement of colorectal SEMS as a bridge therapy for malignant colorectal obstruction with those who had undergone emergency surgery for the same clinical condition. This study was approved by the Ethics and Research Committee of the Cancer Institute of São Paulo Statement (registration number: NP038/14).

Patients with apparently resectable colorectal neoplasia and signs and symptoms of acute colonic obstruction who had undergone emergency surgery or colorectal SEMS placement were included in the study. Patients presenting with signs of an unresectable tumor and those with a metastatic disease for which they were receiving palliative care were excluded.

Before therapeutic interventions, chest and abdominal computed tomography was performed. Patients treated between 2009 and 2013 were referred for endoscopic treatment, whereas those treated between 2014 and 2017 were referred for surgical colostomy.

All endoscopic procedures were performed by the same team with significant experience in oncologic endoscopic procedures, using a combination of endoscopic and fluoroscopic guidance and with patients under general anesthesia. A therapeutic double-lumen gastroscope (Olympus^®^ GIF-2T160) was preferred for these procedures. Endoscopy combined fluoroscopy was used to guide the introduction of SEMS. A straight-line catheter (Tandem™, Boston Scientific) was advanced until the distal border of the malignant stricture. A soft-tip guidewire measuring 0.035 inches (Dreamwire™ Standard, Boston Scientific) was inserted through the catheter under fluoroscopic manipulation until the stricture was trespassed. Uncovered self-expanded metallic stents were deployed under fluoroscopic control with direct endoscopic view of the distal end of the stent. Balloon dilatation was not performed. The colon stents used were WallFlex™, Boston Scientific, Evolution™, Cook Medical and Single Laye™, Hanarostent™, and M.I. Tech. No antibiotic prophylaxis was used. We only used retrograde preparations with low volume (150�200 mL) enema, to prevent the risk of worsening the obstructive condition, as a recommended by the Europeon Society for Gastrointestinal Endoscopy (ESGE) (25).

All surgeries were performed at the same center by a team experienced in colorectal oncology surgeries. Patients showing signs and symptoms of acute colonic obstruction underwent a colostomy to alleviate acute signs. Subsequently, when the patient’s condition improved, elective tumor resection was performed. Patients who initially underwent stent placement may have been treated with surgical colostomy after failure to relieve acute symptoms (clinical success failure).

Technical success was determined by the correct placement of the stent. Clinical success was determined by the colonic decompression with resolution of obstructive symptoms within 72 hours of stent placement.

Incident and AEs were defined per the American Society for Gastrointestinal Endoscopy consensus (26).

Statistical analysis was performed using IBM SPSS Statistics version 22.0 (IBM SPSS^®^, Chicago, US). The Chi-square test was used to compare proportions. The median follow-up time was compared between the groups using the Mann-Whitney test. Survival rates were calculated using the Kaplan-Meier method. To compare the differences between survival curves, the Mantel-Haenszel test was used. A *p*-value <0.05 was considered statistically significant. Relative risks (RRs) and 95% confidence intervals (CIs) were also calculated to compare the proportions.

## RESULTS

**Figure 1:**
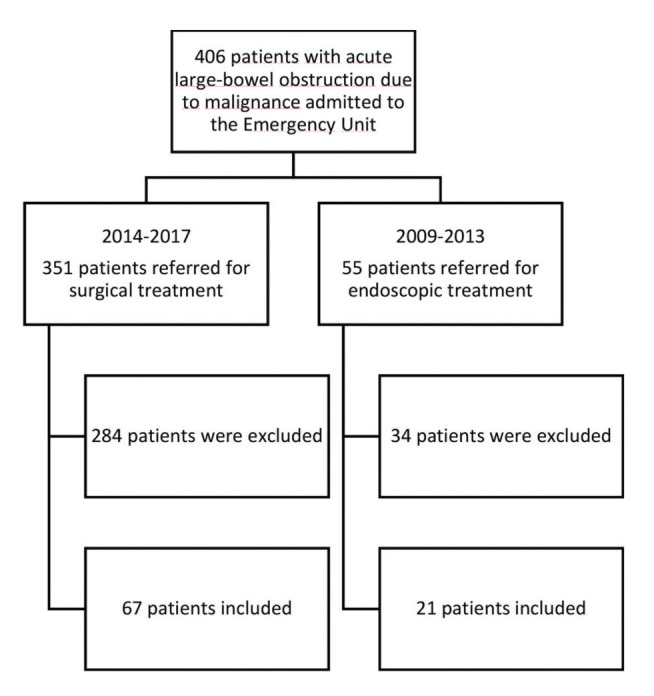
Flowchart of patient selection.

Between 2009 and 2017, 406 consecutive patients were admitted to the emergency room of the hospital with signs of an acute colonic obstruction due to malignant disease (the left colon, sigmoid or rectal cancer). The treatment strategy used (endoscopic or surgical) depended on the period during which the patient was admitted to our institution. Between 2009 and 2013, 56 patients were referred for endoscopic treatment, among whom 35 were excluded because of evidence of unresectable or palliative disease, and the remaining 21 were included in the SEMS group. Between 2014 and 2017, 351 patients were referred for urgent surgical treatment. Similarly, 284 patients were excluded because of evidence of unresectable or palliative disease, and 67 patients were included in the surgical group. (Figure 1).

### Patient characteristics

The majority of patients in the SEMS group were female (57.1%), with a mean age of 59.1 years (range, 36-88 years). In the surgical group, most patients were male (53.7%), with a mean age of 61.6 years (range, 25-92 years) (Table 1).

### Technical success, clinical success, and adverse event rates of endoscopic stenting

Technical and clinical success rates of endoscopic stenting were 95.3% and 85.7%, respectively. Technical failure occurred in one patient, in whom we could not maneuver the guidewire across the stricture. There were no immediate AEs. Severe AEs including perforation, silent perforation, reobstruction, and bleeding were observed in 14.3%, 4.7%, 14.3%, and 14.3% of the patients, respectively. Mild AEs including pain, tenesmus, and incontinence were observed in 42.8%, 9.5%, and 4.76% of the patients, respectively (Table 2).

### Comparison of surgical outcomes in SEMS and emergency surgery groups

The primary surgical outcomes of the SEMS and surgery groups are compared in Table 3. The SEMS group had a higher rate of primary anastomosis (66.6% *vs*. 13.4%; *p*<0.0001) and a lower rate of temporary colostomy (33.3% *vs.* 71.6%; *p*=0.0015) than did the surgery group.

### Long-term analysis of colorectal SEMS *versus* emergency surgery

**Figure 2:**
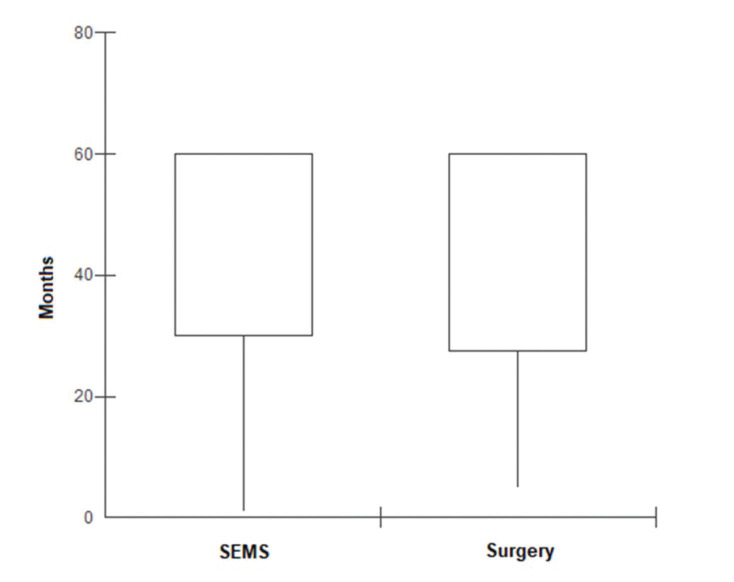
Median follow-up time, interquartile boxplot comparing SEMS and emergency surgery (*p*=0.9337).

**Figure 3:**
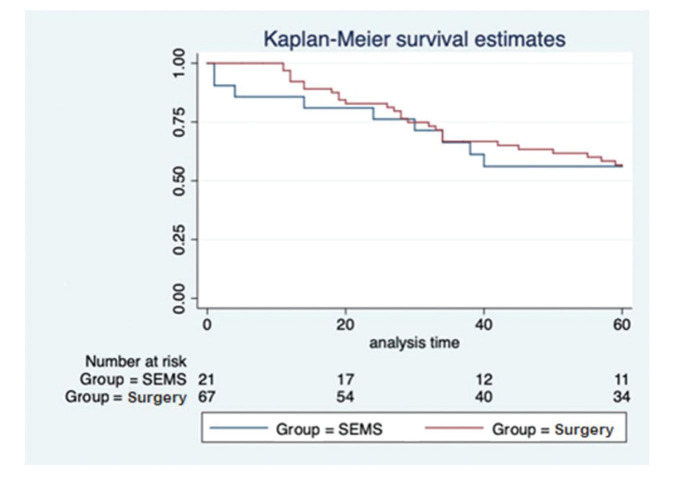
Kaplan-Meier overall survival curve comparing SEMS and emergency surgery.

**Figure 4:**
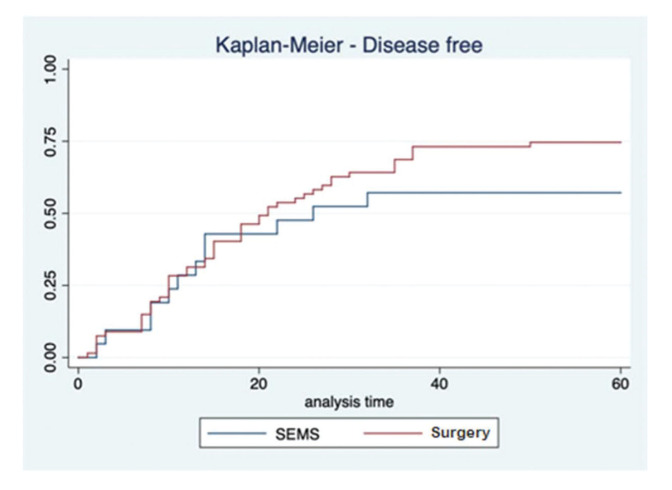
Kaplan-Meier disease-free survival curve comparing SEMS and emergency surgery.

In the SEMS group, the follow-up time ranged from one to 67 months (mean, 41.8±22.1 months). In the emergency surgery group, the minimum follow-up time was five months, whereas the maximum was 69 months (mean, 43.6±19.2 months). These data represent the minimum and maximum number of times patients were monitored in both groups. In the SEMS group, a patient died within a month after the procedure. The same rationale was applied to the surgery group. The median follow-up time was 60 months for both groups, with the same interquartile range of 60 months (*p*=0.9337; Figure 2). During this period of observation, there was no difference in the local recurrence (38.1% *vs*. 22.4%, *p*=0.14) or distant recurrence (33.3% *vs.* 50.7%, *p*=0.16) rates between the SEMS and surgical groups. Further, there was no difference in the overall survival (log rank *p*=0.873) and disease-free survival (log rank *p*=0.2821) rates in the five-year analysis (Figures 3 and 4).

## Discussion

Colorectal cancer patients with acute obstruction may benefit from the application of a colorectal stent as a bridge treatment before surgery. This strategy has been adopted to avoid emergency surgery, which is associated with higher morbidity and mortality (27,28). Stent placement may relieve the obstruction, leading to clinical compensation and adequate preoperative staging. This improves the chances of a single follow-up procedure being sufficient without the need for colostomy. Additionally, this strategy is associated with lower postoperative AE rates. In this study, we observed higher rates of fistula and permanent colostomy in the group that underwent emergency surgery, which might affect the patients’ quality of life. Our results are similar to the findings of several previous meta-analyses (8-10,29).

Hypothetically, SEMS can induce micro-perforation because of the seeding of malignant cells in the peritoneum and other organs. Maruthachalam et al. reported a significant increase in cytokeratin 20 mRNA expression in the peripheral venous blood samples of patients with colorectal cancer who had undergone SEMS placement compared to that in those of the control group. However, the clinical significance of this finding is unknown (30). Haraguchi et al. reported a higher incidence of perineural invasion after using SEMS than that in the control group (59.1% *vs.* 18.2%, *p*=0.0053) (31). This suggests that the interval between the introduction of SEMS and the colostomy should be kept as short as possible, to reduce micro-perforation and late AE rates.

Unfortunately, in more than 40% of our patients (9/21), the time elapsed between SEMS placement was greater than eight weeks. In three patients, this duration was greater than five months. The high late AE rates may be explained by this observation. In addition, four of five patients with local recurrence after colostomy had SEMS permanence longer than six weeks. The risk of local recurrence was four times higher in these patients (RR=4.4, 95% CI=0.58-33.1).

Concerns regarding higher cancer recurrence rates in patients who had undergone colorectal SEMS placement emerged after the results of the first prospective randomized trials with long-term follow-up (4-6,16,32). Since then, the 2014 ESGE guidelines have suggested the use of SEMS as a bridge therapy for palliation of malignant colorectal obstruction only in patients with increased risk of postoperative mortality [American Society of Anesthesiologists (ASA)≥III] and/or older than 70 years. Importantly, the ESGE guidelines have recommended that the use of colorectal stents should be restricted to centers with resources and endoscopists trained and experienced in advanced endoscopy procedures (15). In 2020, the ESGE guidelines were updated, suggesting that the decision regarding the use of stents as a bridge treatment until surgery should involve a multidisciplinary team. In addition, the ESGE suggests that a colonic stent should not be considered if an endoscopist expert is not available to perform the procedure (25). Another concern came to light after 2014 with the publication of the first ESGE guideline: some studies reported a greater risk of locoregional recurrence during long-term follow-up in patients undergoing stent placement. Because of the doubts that arose among experts worldwide at that time, surgical procedures have been preferred since 2014.

When we assessed the results of studies that showed higher cancer recurrence rates in patients who had undergone SEMS placement, the low technical success rates were noticeable (4,5). In the present series, our technical and clinical success rates were considerably high (95% and 86%, respectively). In the Dutch trial by van Hooft et al., failure to deploy the stents was observed in 14 patients (29.8%). Pirlet et al. reported technical failure in 16 patients (53.3%), in addition to three patients with perforation during SEMS insertion (4,5). It is possible that these results were partially due to the inclusion of several nonacademic centers with few technical experts and low expertise in advanced endoscopic procedures. The low technical success rate and the high AE rates reported by those studies may have contributed to the reported high cancer recurrence rates.

In subsequent years, more randomized controlled trials (RCTs) reporting higher technical and clinical success rates were published (33,34). A meta-analysis of RCTs published in 2013 showed a 96.6% technical success rate (35). When the long-term results of these studies were published, the risk of tumor recurrence also seemed to be lower than the initial results. Two meta-analyses, including randomized and non-randomized studies, showed no difference in three- and five-year recurrence and overall survival rates between patients who had undergone SEMS placement and those who had undergone emergency surgery (8,36). In contrast to these findings, a meta-analysis including only RCTs found higher tumor recurrence rates in patients who had undergone SEMS placement (OR=1.79, 95% CI=1.09-2.93, *p*=0.02), but lower stoma, lower complication rates, and higher primary anastomosis rates were found in those patients (29). The robustness of these results may compromise the application of the fixed model to meta-analyze the data, as there was high heterogeneity among the studies (I^2^=53%) (37). Arezzo et al. also published a meta-analysis that included only RCTs, wherein using a random model for meta-analysis they did not find any difference between groups regarding tumor recurrence (RR=1.65, 95% CI=0.95-2.89).

Our study compared the use of SEMS and emergency surgery in potentially treatable patients, demonstrating that there was no difference in the disease-free survival and overall survival rates at the five-year follow-up. Therefore, our results add to the increasing evidence that SEMS placement does not affect patient survival (19-21). In addition, the local and global recurrence rates (local and distant recurrence) did not differ between the groups. The high global recurrence rate found in our study may be explained by the fact that most patients in both groups had T3 or T4 disease and half of them had N1 or N2 disease.

However, this study has several limitations. First, this was a retrospective and single-center study. Second, the selection of the treatment strategy depended on the period during which the patient was admitted to the institution. Despite the obvious selection bias, the groups of comparison were not different, except for a greater frequency of M1 disease in the SEMS group. This difference could have favored the surgical treatment group. Third, this bias could also have influenced the definitive oncological treatment offered to the patients (*e.g.*, novel chemotherapy, robotic surgery). Again, this difference could have favored the results of the patients in the surgery group. Additionally, it is important to determine the definitive treatment as soon as possible. Fourth, the small sample size limited the assessment of predictive factors for five-year survival as well as the measurement of secondary outcomes such as the impact of time elapsed between SEMS placement and colostomy on the risk of local recurrence.

In conclusion, no differences in the disease-free survival and overall survival were observed in the five-year analysis in patients with resectable colorectal cancer who had undergone SEMS placement and colostomy for the treatment of malignant colorectal obstruction. Patients in the SEMS group showed a higher rate of primary anastomosis and a lower rate of permanent colostomy than did those in the surgery group.

## Conflict of Interest

Fauze Maluf-Filho is consultant for Boston Sci, Cook Inc and Olympus Inc. The other authors declare that they have no conflicts of interest.

## AUTHOR CONTRIBUTIONS

Scomparin RC contributed in acquired, analyzed, interpreted the data, drafted the manuscript and provided final approval for publication. Martins BC, Ribeiro Jr U and Nahas SC contributed in analyzed and interpreted the data, critically reviewed the manuscript, and approved the final version for publication. Lenz L, Bento LH, Marques CS, and Safatle-Ribeiro A contributed in acquired, analyzed, interpreted the data and drafted the manuscript. Maluf-Filho F contributed in conceptualized and designed the study, interpreted the data, critically reviewed the manuscript and gave final approval for publication.

## Figures and Tables

**Table 1:** Baseline clinical characteristics of patients in both groups.

	SEMS (%)	Surgery (%)	
Total	21	67	*p*-value
Sex			0.3843
Male	9 (42.8%)	36 (53.7%)	
Female	12 (57.1%)	31 (46.2%)	
Mean Age (range)	59.1 (36-88)	61.6 (25-92)	
Tumor localization			0.8242
Descending colon	5 (23.8%)	15 (22.38%)	
Sigmoid colon	6 (28.5%)	24 (35.82%)	
Rectum	10 (47.6%)	28 (41.8%)	
ASA			0.3387
1	4 (19%)	24 (35.6%)	
2	14 (66.6%)	34 (50.5%)	
3	3 (14.2%)	9 (14.3%)	
ECOG			0.9120
0	6 (28.6%)	15 (22.3%)	
1	11 (52.3%)	32 (47.7%)	
2	4 (19%)	15 (22.3%)	
3	0	5 (7.4%)	
T status (TNM)			0.4115
T2	3 (14.2%)	2 (2.9%)	
T3	11 (52.3%)	44 (65.6%)	
T4	7 (33.3%)	21 (31.3%)	
N status (TNM)			0.4977
N0	10 (47.6%)	28 (41.7%)	
N1	5 (23.8%)	25 (37.3%)	
N2	6 (28.6%)	14 (20.8%)	
M status (TNM)			0.0408
M0	18 (85.7%)	66 (98.5%)	
M1	3 (14.3%)	1 (1.5%)	
Previous chemotherapy	17 (80.9%)	17 (25%)	
Previous radiotherapy	10 (47.6%)	18 (27%)	
Days for curative surgery	8 (1-169)	31 (1-2068)	0.02

ASA = American Society of Anesthesiologists Score; ECOG = Eastern Cooperative Oncology Group � Performance Status Score; SEMS = Colorectal Self-Expanding Metal Stents; TNM = Classification of Malignant Tumors.

**Table 2:** Technical success, clinical success, and adverse event rates of endoscopic stenting.

Characteristics	N (%)
Technical success	20 (95.3%)
Clinical success	18 (85.7%)
Immediate adverse events	0
Severe adverse events	
Migration	0
Perforation	3 (14.3%)
Silent perforation	1 (4.7%)
Reobstruction	3 (14.3%)
Bleeding	3 (14.3%)
Mild adverse events	
Pain	9 (42.8%)
Tenesmus	2 (9.5%)
Incontinence	1 (4.7%)

N=Number of patients.

**Table 3:** Comparison of surgical outcomes in SEMS *vs.* surgery groups.

	SEMS=21	Surgery=67	p-Value	RR (95% CI)
Primary anastomosis	14 (66.6%)	9 (13.4%)	<0.0001	4.9630 (2.51 - 9.78)
Temporary colostomy	7 (33.3%)	48 (71.6%)	0.00161	0.4653 (0.24 - 0.86)
Permanent colostomy	3 (14.2%)	24 (35.8%)	0.1001	0.3988 (0.13 - 1.19)
Fistula	1 (4.7%)	10 (14.9%)	0.2620	0.3190 (0.04 - 2.35)
Local recurrence	5 (23.8%)	15 (22.4%)	0.1391	0.8916 (0.43 - 2.57)
Distant recurrence	7 (33.3%)	34 (50.7%)	0.2024	0.6569 (0.34 - 1.25)
Global recurrence	12 (57.1%)	49 (73.1%)	0.8802	0.2241 (0.52 - 1.16)

RR=Relative Risk.

## References

[1] Kuipers EJ, Grady WM, Lieberman D, Seufferlein T, Sung JJ, Boelens PG (2015 Nov 5). Colorectal cancer. Nat Rev Dis Primers.

[2] Haggar FA, Boushey RP (2009). Colorectal Cancer Epidemiology: Incidence, Mortality, Survival, and Risk Factors. Clin Colon Rectal Surg.

[3] Deans GT, Krukowski ZH, Irwin ST (1994). Malignant obstruction of the left colon. Br J Surg.

[4] van Hooft JE, Bemelman WA, Oldenburg B, Marinelli AW, Lutke Holzik MF, Grubben MJ (2011). Colonic stenting versus emergency surgery for acute left-sided malignant colonic obstruction: a multicentre randomised trial. Lancet Oncol.

[5] Pirlet IA, Slim K, Kwiatkowski F, Michot F, Millat BL (2011). Emergency preoperative stenting versus surgery for acute left-sided malignant colonic obstruction: a multicenter randomized controlled trial. Surg Endosc.

[6] Alcántara M, Serra-Aracil X, Falcó J, Mora L, Bombardó J, Navarro S (2011). Prospective, Controlled, Randomized Study of Intraoperative Colonic Lavage Versus Stent Placement in Obstructive Left-sided Colonic Cancer. World J Surg.

[7] Cheung HY, Chung CC, Tsang WW, Wong JC, Yau KK, Li MK (2009). Endolaparoscopic Approach vs Conventional Open Surgery in the Treatment of Obstructing Left-Sided Colon Cancer: a randomized controlled trial. Arch Surg.

[8] Ceresoli M, Allievi N, Coccolini F, Montori G, Fugazzola P, Pisano M (2017). Long-term oncologic outcomes of stent as a bridge to surgery versus emergency surgery in malignant left side colonic obstructions: a meta-analysis. J Gastrointest Oncol.

[9] Arezzo A, Passera R, Lo Secco G, Verra M, Bonino MA, Targarona E (2017). Stent as bridge to surgery for left-sided malignant colonic obstruction reduces adverse events and stoma rate compared with emergency surgery: results of a systematic review and meta-analysis of randomized controlled trials. Gastrointest Endosc.

[10] Huang X, Lv B, Zhang S, Meng L (2014). Preoperative Colonic Stents Versus Emergency Surgery for Acute Left-Sided Malignant Colonic Obstruction: A Meta-analysis. J Gastrointest Surg.

[11] Sagar J (2011). Colorectal stents for the management of malignant colonic obstructions. Cochrane Database Syst Rev.

[12] Watt AM, Faragher IG, Griffin TT, Rieger NA, Maddern GJ (2007). Self-expanding Metallic Stents for Relieving Malignant Colorectal Obstruction: a systematic review. Ann Surg.

[13] Khot UP, Lang AW, Murali K, Parker MC (2002). Systematic review of the efficacy and safety of colorectal stents. Br J Surg.

[14] Sebastian S, Johnston S, Geoghegan T, Torreggiani W, Buckley M (2004). Pooled Analysis of the Efficacy and Safety of Self-Expanding Metal Stenting in Malignant Colorectal Obstruction. Am J Gastroenterol.

[15] van Hooft JE, van Halsema EE, Vanbiervliet G, Beets-Tan RG, DeWitt JM, Donnellan F (2014). Self-expandable metal stents for obstructing colonic and extracolonic cancer: European Society of Gastrointestinal Endoscopy (ESGE) Clinical Guideline. Gastrointest Endosc.

[16] Tung KL, Cheung HY, Ng LW, Chung CC, Li MK (2013). Endo-laparoscopic approach versus conventional open surgery in the treatment of obstructing left-sided colon cancer: Long-term follow-up of a randomized trial. Asian J Endosc Surg.

[17] Gorissen KJ, Tuynman JB, Fryer E, Wang L, Uberoi R, Jones OM (2013). Local recurrence after stenting for obstructing left-sided colonic cancer. Br J Surg.

[18] Sabbagh C, Browet F, Diouf M, Cosse C, Brehant O, Bartoli E (2013). Is Stenting as “a Bridge to Surgery” an Oncologically Safe Strategy for the Management of Acute, Left-Sided, Malignant, Colonic Obstruction? A Comparative Study With a Propensity Score Analysis. Ann Surg.

[19] Ribeiro I, Pinho R, Leite M, Proença L, Silva J, Ponte A (2016). Reevaluation of Self-Expanding Metal Stents as a Bridge to Surgery for Acute Left-Sided Malignant Colonic Obstruction: Six Years Experience. GE Port J Gastroenterol.

[20] Gibor U, Perry Z, Tirosh D, Netz U, Rosental A, Fich A (2017). Comparison of the Long-Term Oncological Outcomes of Stent as a Bridge to Surgery and Surgery Alone in Malignant Colonic Obstruction. Isr Med Assoc J.

[21] Verstockt B, Van Driessche A, De Man M, van der Spek P, Hendrickx K, Casneuf (2018). Ten-year survival after endoscopic stent placement as a bridge to surgery in obstructing colon cancer. Gastrointest Endosc.

[22] Yang SY, Park YY, Han YD, Cho MS, Hur H, Min BS (2019). Oncologic Outcomes of Self-Expandable Metallic Stent as a Bridge to Surgery and Safety and Feasibility of Minimally Invasive Surgery for Acute Malignant Colonic Obstruction. Ann Surg Oncol.

[23] Crespí-Mir A, Romero-Marcos JM, de la Llave-Serralvo A, Dolz-Abadía C, Cifuentes-Ródenas JA (2018). Impact on Surgical and Oncological Results of the Use of Colonic Stents as a Bridge to Surgery for Potentially Curable Occlusive Colorectal Neoplasms. Cir Esp.

[24] Lara-Romero C, Vilches Á, Caunedo-Álvarez Á, Hergueta-Delgado P, Lavín-Castejón I, Andrade-Bellido R (2019). Better recurrence-free survival after stent bridge to surgery compared to emergency surgery for obstructive left-sided colonic cancer in patients with stage III status of the American Joint Committee on Cancer (AJCC): a bicentric retrospective study. Int J Colorectal Dis.

[25] van Hooft JE, Veld JV, Arnold D, Beets-Tan RGH, Everett S, Götz M (2020). Self-expandable metal stents for obstructing colonic and extracolonic cancer: European Society of Gastrointestinal Endoscopy (ESGE) Guideline - Update 2020. Endoscopy.

[26] Cotton PB, Eisen GM, Aabakken L, Baron TH, Hutter MM, Jacobson BC (2010). A lexicon for endoscopic adverse events: report of an ASGE workshop. Gastrointest Endosc.

[27] Scott NA, Jeacock J, Kingston RD (1995). Risk factors in patients presenting as an emergency with colorectal cancer. Br J Surg.

[28] Watt AM, Faragher IG, Griffin TT, Rieger NA, Maddern GJ (2007). Self-expanding metallic stents for relieving malignant colorectal obstruction: a systematic review. Ann Surg.

[29] Yang P, Lin XF, Lin K, Li W (2018). The Role of Stents as Bridge to Surgery for Acute Left-Sided Obstructive Colorectal Cancer: Meta-Analysis of Randomized Controlled Trials. Rev Invest Clin.

[30] Maruthachalam K, Lash GE, Shenton BK, Horgan AF (2007). Tumour cell dissemination following endoscopic stent insertion. Br J Surg.

[31] Haraguchi N, Ikeda M, Miyake M, Yamada T, Sakakibara Y, Mita E (2016). Colonic stenting as a bridge to surgery for obstructive colorectal cancer: advantages and disadvantages. Surg Today.

[32] Sloothaak DAM, van den Berg MW, Dijkgraaf MGW, Fockens P, Tanis PJ, Hooft JEV (2013). Recurrences after endoscopic stenting as treatment for acute malignant colonic obstruction in the Dutch Stent-In 2 trial. UEG Week.

[33] Ghazal AH, El-Shazly WG, Bessa SS, El-Riwini MT, Hussein AM l (2013). Colonic Endolumenal Stenting Devices and Elective Surgery Versus Emergency Subtotal/Total Colectomy in the Management of Malignant Obstructed Left Colon Carcinoma. J Gastrointest Surg.

[34] Ho KS, Quah HM, Lim JF, Tang CL, Eu KW (2012). Endoscopic stenting and elective surgery versus emergency surgery for left-sided malignant colonic obstruction: a prospective randomized trial. Int J Colorectal Dis.

[35] De Ceglie A, Filiberti R, Baron TH, Ceppi M, Conio M (2013). A meta-analysis of endoscopic stenting as bridge to surgery versus emergency surgery for left-sided colorectal cancer obstruction. Crit Rev Oncol Hematol.

[36] Amelung FJ, Burghgraef TA, Tanis PJ, van Hooft JE, Ter Borg F, Siersema PD (2018). Critical appraisal of oncological safety of stent as bridge to surgery in left-sided obstructing colon cancer; a systematic review and meta-analysis. Crit Rev Oncol Hematol.

[37] Arezzo A, Lo Secco G, Passera R, Morino M (2019). Response. Gastrointest Endosc.

